# Design, synthesis and biological evaluation of novel O-substituted tryptanthrin oxime derivatives as c-Jun *N*-terminal kinase inhibitors

**DOI:** 10.3389/fphar.2022.958687

**Published:** 2022-09-12

**Authors:** Igor A. Schepetkin, Anastasia R. Kovrizhina, Ksenia S. Stankevich, Andrei I. Khlebnikov, Liliya N. Kirpotina, Mark T. Quinn, Matthew J. Cook

**Affiliations:** ^1^ Department of Microbiology and Cell Biology, Montana State University, Bozeman, MT, United States; ^2^ Kizhner Research Center, Tomsk Polytechnic University, Tomsk, Russia; ^3^ Department of Chemistry and Biochemistry, Montana State University, Bozeman, MT, United States

**Keywords:** anti-inflammatory, 11H-indeno[1,2-b]quinoxalin-11-one, interleukin-6, c-Jun N-terminal kinase, nuclear factor-κB, oxime, tryptanthrin

## Abstract

The c-Jun *N*-terminal kinase (JNK) family includes three proteins (JNK1-3) that regulate many physiological processes, including inflammatory responses, morphogenesis, cell proliferation, differentiation, survival, and cell death. Therefore, JNK represents an attractive target for therapeutic intervention. Herein, a panel of novel tryptanthrin oxime analogs were synthesized and evaluated for JNK1-3 binding (K_d_) and inhibition of cellular inflammatory responses (IC_50_). Several compounds exhibited submicromolar JNK binding affinity, with the most potent inhibitor being 6-(acetoxyimino)indolo[2,1-*b*]quinazolin-12(6*H*)-one (**1j**), which demonstrated high JNK1-3 binding affinity (K_d_ = 340, 490, and 180 nM for JNK1, JNK2, and JNK3, respectively) and inhibited lipopolysaccharide (LPS)-induced nuclear factor-κB/activating protein 1 (NF-κB/AP-1) transcription activity in THP-1Blue cells and interleukin-6 (IL-6) production in MonoMac-6 monocytic cells (IC_50_ = 0.8 and 1.7 μM, respectively). Compound **1j** also inhibited LPS-induced production of several other proinflammatory cytokines, including IL-1α, IL-1β, granulocyte-macrophage colony-stimulating factor (GM-CSF), monocyte chemoattractant protein-1 (MCP-1), and tumor necrosis factor (TNF) in MonoMac-6 cells. Likewise, **1j** inhibited LPS-induced c-Jun phosphorylation in MonoMac-6 cells, directly confirming JNK inhibition. Molecular modeling suggested modes of binding interaction of selected compounds in the JNK3 catalytic site that were in agreement with the experimental JNK3 binding data. Our results demonstrate the potential for developing anti-inflammatory drugs based on these nitrogen-containing heterocyclic systems.

## 1 Introduction

*c*-Jun *N*-terminal kinases (JNKs) are members of the mitogen-activated protein kinase (MAPK) family and mediate eukaryotic cell responses to abiotic and biotic stress (Kyriakis et al., 1994). The JNK pathway is a highly complex cassette within the MAPK signaling network (Johnson and Lapadat, 2002). JNK activation can be induced by other MAPKs, as well as G protein-coupled receptors (GPCRs), which feed information into the JNK signaling pathway. Moreover, it has been demonstrated that JNKs can undergo significant autophosphorylation (Vogel et al., 2009; Wang et al., 2020). Despite the name, activated JNKs can phosphorylate an number of proteins in addition to *c*-Jun, with close to 100 protein substrates known to date (Hammouda et al., 2020). Specific JNK3 substrates have also been identified, including voltage-dependent anion channel (VDAC) (Gupta and Ghosh, 2015; Gupta, 2017; Gupta and Ghosh, 2017). Nevertheless, *c*-Jun is a major substrate for JNKs, and its phosphorylation is closely tied to activator protein 1 (AP-1) activation.

The human genome contains three closely related genes (JNK1, JNK2, and JNK3), with each gene encoding multiple isoforms (Ha et al., 2019). JNK1 and JNK2 are expressed in a wide variety of tissues throughout the body, whereas JNK3 is mainly expressed in neurons and to a lesser extent in the heart and testes (Bode and Dong, 2007). Although the structure and sequences of all JNKs are similar, containing well conserved features observed in other MAPKs, JNK1 and JNK3 closely resemble each other, with JNK2 containing sequence differences in the kinase domain (Gupta et al., 1996). Despite the multifaceted role of JNKs in cell signaling, JNK has still been viewed as a promising target for several disease areas (Lai et al., 2020; Yung and Giacca, 2020; Abdelrahman et al., 2021). Our interest comes from its role in mediating inflammatory and immunological responses (Lai et al., 2020; Chen et al., 2021). Indeed, JNK inhibition has been demonstrated to downregulate the production of several proinflammatory transcription factors and cytokines (Kaminska et al., 2009; Lai et al., 2020). Due to the physiological isoform distribution within the body, therapeutic selectivity between the JNK isoforms could have major therapeutic benefits. JNK1/2 are attractive targets for treating chronic inflammatory diseases, such as rheumatoid arthritis, whereas JNK3 could be a promising target for treating neuroinflammation (Mehan et al., 2011; Kersting et al., 2013; Cubero et al., 2015).

**FIGURE 1:**
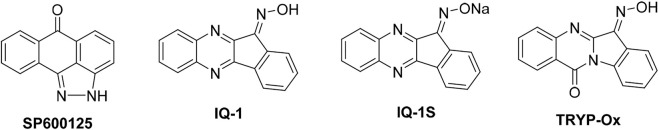
Chemical structures of several JNK inhibitors.

Due to the potential clinical significance of the JNKs, many groups have sought to develop inhibitors for these targets (Gehringer et al., 2015; Cho and Hah, 2021). The first potent inhibitor reported was SP600125 (Figure 1), which exhibited pan-JNK activity but also inhibited numerous other MAPKs (Bennett et al., 2001). We examined other heterocyclic structures and found that derivatives of indeno[1,2-*b*]quinoxalinone oxime (**IQ-1**) were both potent and selective (Figure 1) (Schepetkin et al., 2012; Schepetkin et al., 2019). Indeed, these **IQ-1** derivatives were equipotent with SP600125 and had much higher selectivity profiles, with significant binding only seen at CK1δ and JNK1-3 when tested against a panel of kinases (Schepetkin et al., 2012; Schepetkin et al., 2019). Additionally, these compounds were shown to downregulate proinflammatory responses in human monocytic cell lines and in animal models (Schepetkin et al., 2012; Schepetkin et al., 2015; Schepetkin et al., 2019; Kirpotina et al., 2020; Seledtsov et al., 2020). More recently, we discovered that other planar heterocyclic core structures could provide similar and complementary activity, with oxime derivatives of the natural product tryptanthrin (**TRYP-Ox**) proving especially effective (Schepetkin et al., 2019; Kirpotina et al., 2020). Herein, we report our investigation of the effect that chemical modification of the oxime group has on the biological activity of the **TRYP-Ox** scaffold. The premise of this study was to determine the role of the oxime functionality in enzymatic binding, cellular mediation of inflammatory responses, and phosphorylation of *c*-Jun.

## 2 Results and discussion

### 2.1 Chemistry

**SCHEME 1:**
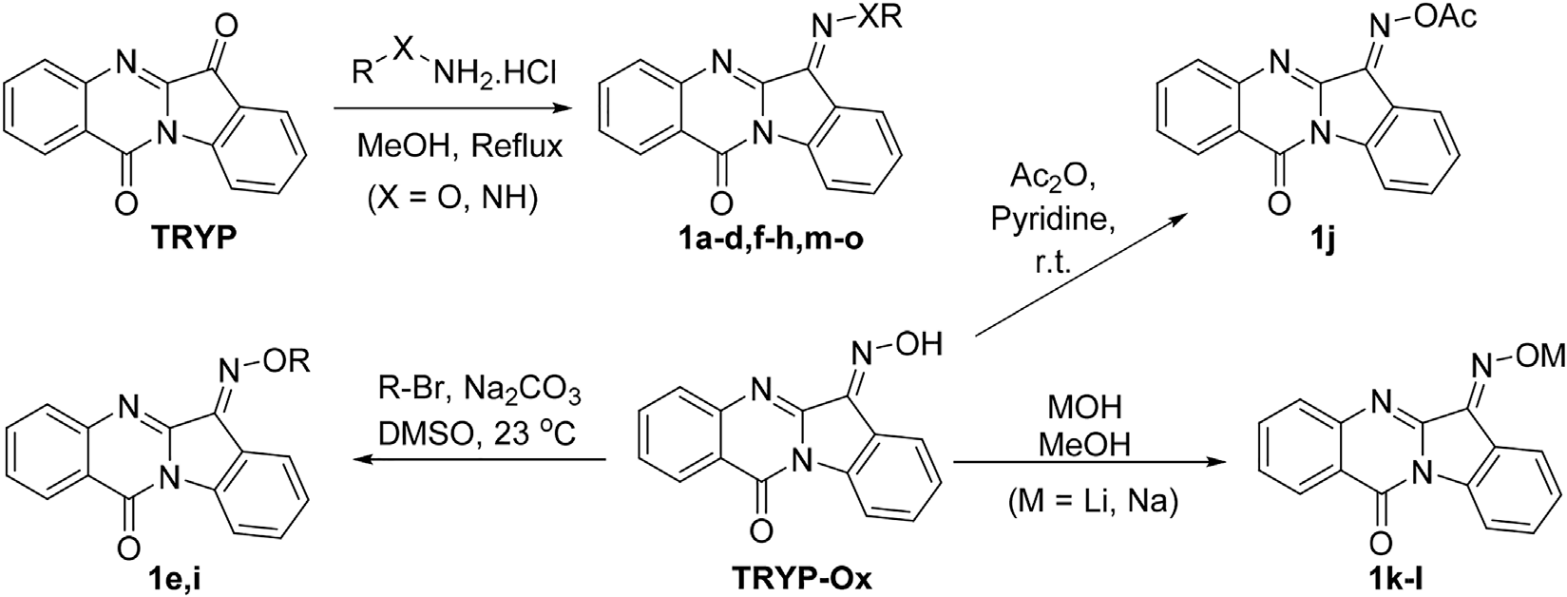
Synthetic routes to tryptanthrin derivatives.

Our previous studies on indenoquinoxaline and tryptanthrin structures demonstrated that the oxime was crucial for JNK inhibition (Schepetkin et al., 2012; Schepetkin et al., 2019). We therefore examined the effect that substitution of the oxime OH had on JNK inhibitory activity. To accomplish this, we synthesized a range of tryptanthrin derivatives where the oxime group was replaced (**1a**-**o**). The synthesis of these compounds was achieved from tryptanthrin (**TRYP**) or the previously reported **TRYP-Ox** (Scheme 1). Substituted oximes and hydrazones were synthesized through direct condensation of the corresponding hydroxylamine ether, hydrazine, or semicarbazide with **TRYP**. Alternatively, **TRYP-Ox** could undergo base-mediated alkylation or acylation to provide the *O*-alkyl and *O*-acyl derivatives. Lithium and sodium salts were obtained through treatment with LiOH and NaOH, respectively. All compounds were obtained as mixtures of *E*/*Z* isomers; however, the interconversion of (*E*/*Z*) oximes and oxime ethers in solution is well established (Diethelm-Varela et al., 2021).

### 2.2 Evaluation of compound biological activity

Prior to evaluation in both enzymatic and cellular assays, we measured the cytotoxicity of compounds **1a-o** in human monocytic THP-1Blue and MonoMac-6 cells following a 24 h incubation. All compounds reported in this study had no effect on cell viability at concentrations up to 50 μM (data not shown), which is similar to the lack of toxicity observed for the parent compounds (Schepetkin et al., 2019; Kirpotina et al., 2020). The compounds were evaluated for their ability to bind to JNK1-3 and compared with previously reported **TRYP-Ox**, a JNK inhibitor (Schepetkin et al., 2019). We used the KINOMEscan ATP site–dependent binding assay, which reflects biologically relevant behavior of the kinases (Fabian et al., 2005; Karaman et al., 2008). The JNK pathway can be activated through Toll-like receptor 4 (TLR4), leading to the activation of transcription factors NF-κB and AP-1 [reviewed in (Aggarwal, 2000; Guha and Mackman, 2001; Takeuchi and Akira, 2001)]. Thus, to assess the anti-inflammatory activity of test derivatives, compounds **1a-o** were also evaluated for their ability to inhibit lipopolysaccharide (LPS)-induced NF-κB/AP-1 reporter activity and IL-6 production in THP-1Blue and MonoMac-6 cells, respectively (Schepetkin et al., 2012).

In particular, *O*-alkyl substituted oximes had little to no JNK binding affinity and did not inhibit cellular anti-inflammatory responses. *O*-Acyl groups, however, provided very similar JNK binding affinities to the parent oxime, with increased cellular anti-inflammatory activity. This suggested that the ester C-O bond was cleaved in both assays with substitution increasing cellular uptake. The aim of this study was to investigate the effect oxime group modification of the tryptanthrin oxime scaffold had on both JNK binding and cellular activity. Importantly, these results will help determine whether the two structures share a common binding epitope and what, if any, key differences in structure–activity relationship (SAR) exist between the two series.

Our baseline for biological activity was the unsubstituted oxime (**TRYP-Ox**), which exhibited pan-JNK binding with JNK-1/3 selectivity over JNK-2. Additionally, **TRYP-Ox** inhibited cytokine production in LPS-stimulated THP-1Blue and MonoMac-6 cells with IC_50_ values of 3.8 and 3.2 μM, respectively (Schepetkin et al., 2019). Upon *O*-alkylation, the biological profile of these molecules changed, with increased JNK-3 selectivity, albeit with decreased binding affinity. Simple alkyl groups (**1a-d**) gave moderate JNK-3 selectivity with 2-4-fold higher binding over JNK-1 and 4-6-fold over JNK-2 (Table 1). Additionally, these compounds were poorly active (or inactive) in the cellular assay, suggesting poor cellular uptake and/or microsomal instability within the cells. This selectivity trend is even more pronounced in methylene nitrile ether **1e**, which showed >10-fold discrimination for JNK-3 over JNK-1/2, again with no cellular activity. *O*-Benzyl oxime ethers (**1f**-**h**) were inactive, whereas 2-pyridyl ether (**1i**) exhibited moderate JNK activity and selectivity, demonstrating the tolerance of aromatic groups when a basic group was present. Similarly to the **IQ-1** series (Schepetkin et al., 2012), acylated oxime (**1j**) provided comparable enzymatic activity to **TRYP-Ox** but with enhanced anti-inflammatory activity. Likewise, lithium and sodium oxime salts (**1k**,**l**) gave similar profiles to **TRYP-Ox** but with more potent cellular function. The dichotomy between the enzymatic and cellular data for these three compounds (**2j-l**) compared to **TRYP-Ox** suggested that their increased solubility led to increased cellular uptake; however, the active ligand was still **TRYP-Ox**. Indeed, the measured p*K*_*a*_ range of diaryl oximes is 8–11, leading to rapid protonation in pH 7.4 buffer solution (Mangold et al., 1989). Hydrazone derivatives (**1m-n**) were poorly soluble and inactive in cellular assays. Semicarbazone variant (**1o**) had a much better solubility profile, exhibiting sub-micromolar binding affinities with all JNK isoforms tested and moderate inhibition of anti-inflammatory activity.

**TABLE 1:** Binding affinity (K_d_) of compounds **1a-o** and their inhibitory effect on LPS-induced NF-κB/AP-1 transcriptional activity in THP-1Blue cells and IL-6 production in MonoMac-6 cells.

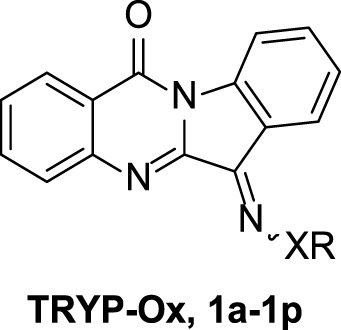						
Compd.	XR	Binding affinity			THP-1Blue NF-κB/AP-1	MonoMac-6 IL-6 production
		JNK1	JNK2	JNK3		
	K_d_ (μM)			IC_50_ (μM)	
TRYP-Ox^a^	-OH	0.15 ± 0.081^a^	1.0 ± 0.14^a^	0.275 ± 0.21^a^	3.8 ± 1.1^a^	3.2 ± 1.2^a^
1a	-OCH_3_	3.1 ± 0.8	5.6 ± 0.4	1.4 ± 0.2	20.8 ± 5.3	23.5 ± 1.8
1b	-OC_2_H_5_	3.2 ± 1.2	5.4 ± 0.6	1.2 ± 0.4	N.A.	N.A.
1c	-OCH_2_CH=CH_2_	3.6 ± 0.7	4.1 ± 0.1	0.85 ± 0.01	18.9 ± 1.8	N.A.
1d	-OC(CH_3_)_3_	9.3 ± 0.5	12.0 ± 1.4	2.3 ± 0.3	N.A.	N.A.
1e	OCH_2_CN	19.5 ± 3.5	17.5 ± 2.1	1.5 ± 0.6	N.A.	N.A.
1f	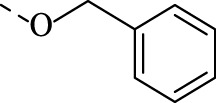	n.d.	n.d.	N.B.	N.A.	N.A.
1g	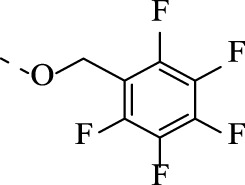	n.d.	n.d.	N.B.	N.A.	N.A.
1h	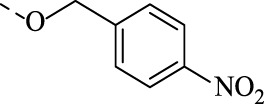	Poor solubility^b^			N.A.	N.A.
1i	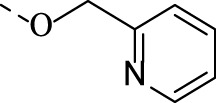	4.0 ± 0.9	3.9 ± 0.4	1.5 ± 0.1	N.A.	N.A.
1j	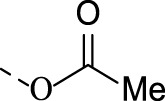	0.34 ± 0.04	0.49 ± 0.01	0.18 ± 0.04	0.8 ± 0.2	1.7 ± 0.1
1k	-OLi	0.47 ± 0.03	0.62 ± 0.035	0.17 ± 0.03	0.9 ± 0.1	1.1 ± 0.2
1l	-ONa	0.44 ± 0.07	0.58 ± 0.014	0.22 ± 0.04	0.9 ± 0.2	1.8 ± 0.2
1m	-NH_2_	Poor solubility^b^			N.A.	N.A.
1n	-NHPh	Poor solubility^b^			N.A.	N.A.
1o	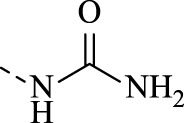	0.47 ± 0.03	0.62 ± 0.035	0.17 ± 0.03	3.3 ± 0.1	6.6 ± 1.2

a Data for from (Schepetkin et al., 2019).

b Compound was insufficiently soluble in dimethyl sulfoxide (DMSO) for the binding affinity assay.

N.A., no inhibition at concentrations ≤ 50 μM; n.d., not determined; N.B., no JNK binding at concentrations ≤ 30 μM.

When comparing the enzymatic and cellular data from the **TRYP-Ox** series to the **IQ-1** series (Schepetkin et al., 2012), there was a clear difference. Specifically, *O*-substitution was much better tolerated in the tryptanthrin analogs, with alkyl groups still providing moderate JNK-binding and selectivity, although aromatic substituted alkyl groups were inactive in the absence of a basic group. The effect of acetyl substitution (**1j**) and deprotonation (**1k**,**j**) mirrored that of **IQ-1** and **IQ-1S**, with similar levels of binding affinity and higher efficacy in the cellular assays (Mangold et al., 1989; Schepetkin et al., 2012). This similar trend further supports the hypothesis that the increased cellular activity is due to higher solubility and/or cellular permeability.

**FIGURE 2:**
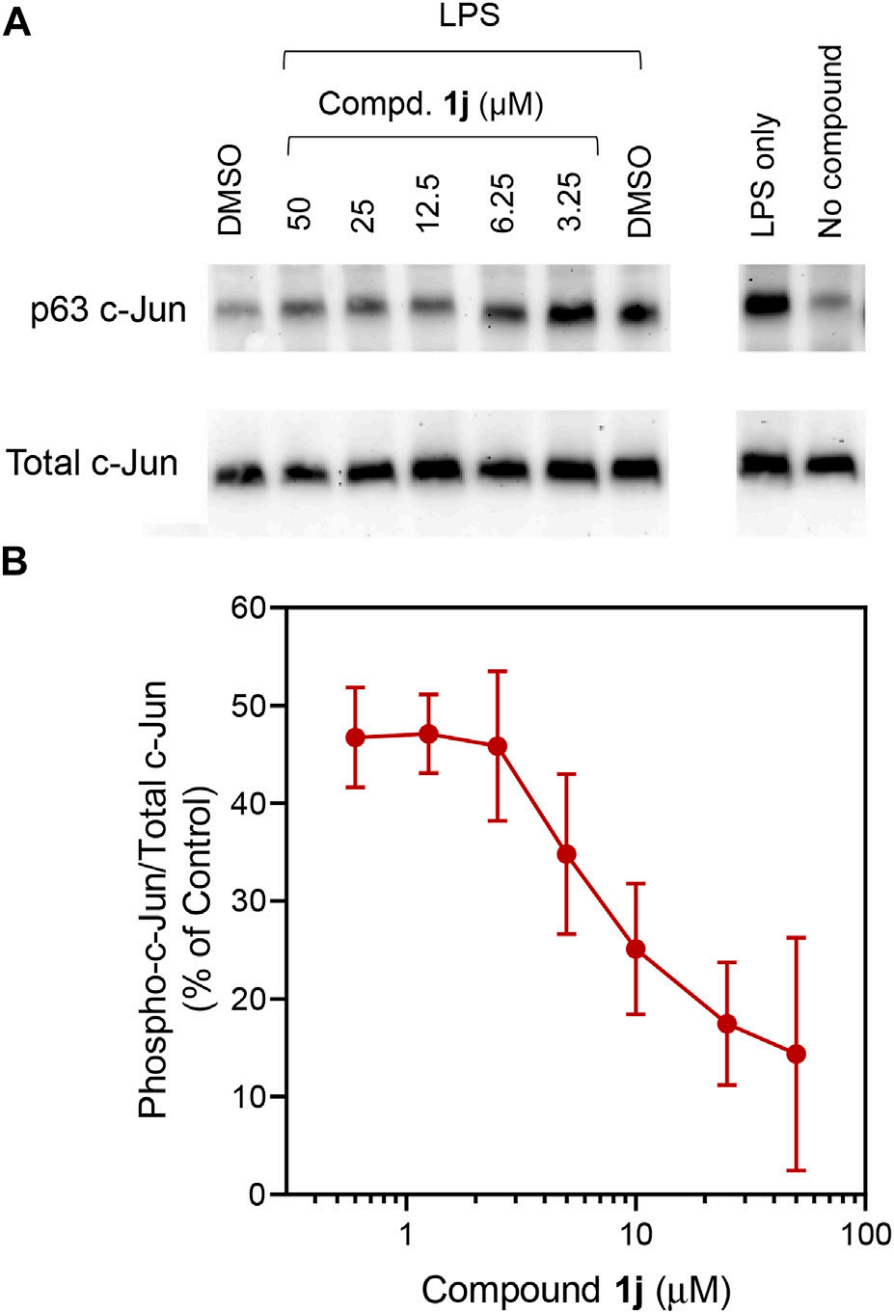
Effect of **1j** on LPS-induced c-Jun (Ser63) phosphorylation. Human MonoMac-6 monocytic cells were pretreated with the indicated concentrations of compounds or 0.5% DMSO for 30 min, followed by treatment with LPS (250 ng/ml) or control buffer for another 30 min. Controls with cells alone (no DMSO) or cells treated with LPS alone were also included. The cells were lysed, and the lysates were analyzed by Western blotting on 10% SDS-PAGE gels. A representative blot from three independent experiments is shown **(A)**. The blot was analyzed by densitometry as described under *Materials and Methods*, and the ratio of phospho-*c*-Jun/total *c*-Jun is shown in **(B)**. Values are expressed as mean ± SD of three independent experiments.

**FIGURE 3:**
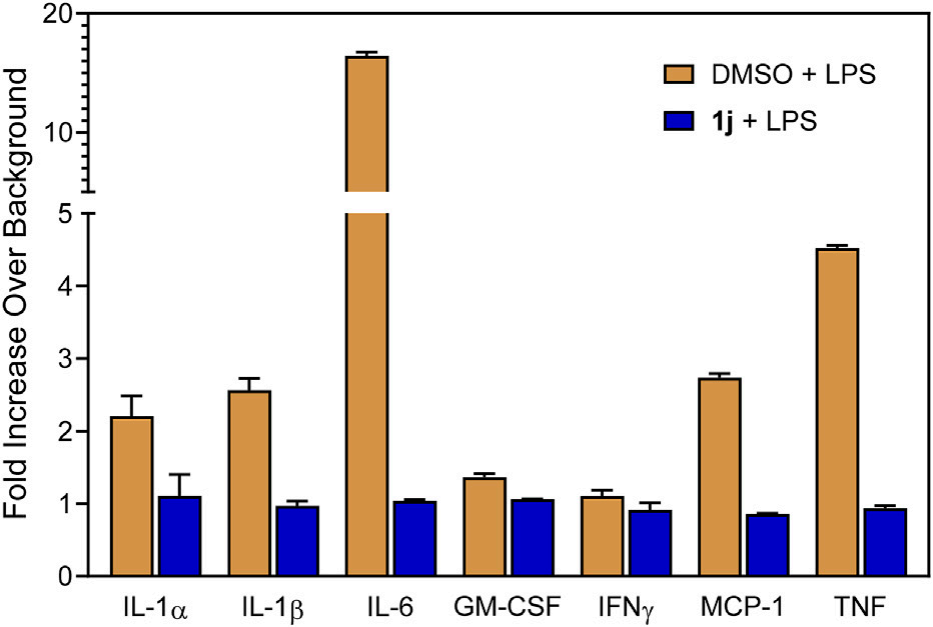
Effect of the compound **1j** on proinflammatory cytokine production in MonoMac-6 cells. Human MonoMac-6 monocytic cells were pretreated with 10 μM of **1j** or DMSO for 30 min, followed by addition of 250 ng/ml LPS or buffer for 24 h. Production of cytokines in the supernatants was evaluated using a Multiplex human cytokine ELISA kit. The relative level of cytokine production is shown as fold increase over background (i.e., 1% DMSO control). The data are presented as the mean ± S.D. of triplicate samples from one experiment that is representative of two independent experiments.

In order to probe whether the increased anti-inflammatory activity observed for **1j** was a result of JNK inhibition, we evaluated its effect on *c*-Jun phosphorylation in MonoMac-6 monocytic cells. These cells were pretreated with the compounds, stimulated with LPS (250 ng/ml), and the level of phospho-*c*-Jun (S63) was determined (Figure 2A). Although phosphorylation can occur at S63/73 and T91/93 (Morton et al., 2003; Vinciguerr et al., 2008; Madeo et al., 2010), S63 activation occurs in all cases. Therefore, anti-phosphoS63 antibody was selected and benchmarked against total *c*-Jun. Indeed, we saw a dose-dependent inhibition of phosphorylation (Figure 2B) similar to our previous studies with **TRYP-Ox** (Schepetkin et al., 2019), providing further evidence for the increased cell permeability and conversion to **TRYP-Ox**. The effect that compound **1j** had on production of proinflammatory cytokines was investigated using a Multiplex human cytokine ELISA kit against seven different cytokines and chemokines. Compound **1j**, at a concentration of 10 μM, completely inhibited the secretion of IL-1α, IL-1β, IL-6, tumor necrosis factor (TNF), monocyte chemoattractant protein-1 (MCP-1), and granulocyte-macrophage colony-stimulating factor (GM-CSF) in LPS-stimulated MonoMac-6 cells compared with dimethyl sulfoxide (DMSO)-treated control cells. The effect on interferon-γ (IFN-γ) production was inconclusive because of low production of this cytokine after LPS treatment of MonoMac-6 cells (Figure 3). These data further demonstrate the anti-inflammatory activity of **1j**.

### 2.3 Molecular modeling

**FIGURE 4:**
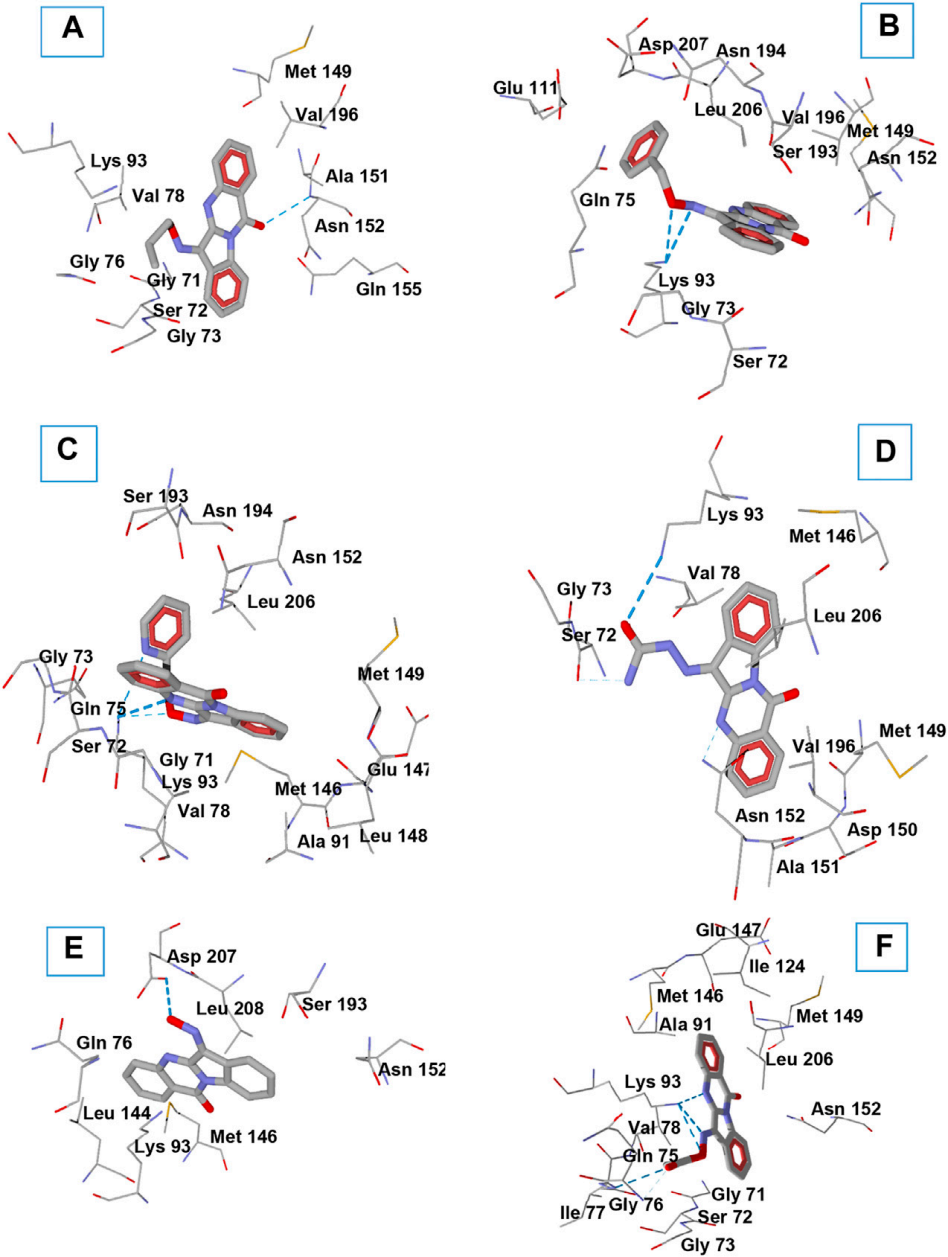
Docking poses of compounds **1c (A)**, **1f (B)**, **1i (C)**, **1o (D)**, **TRYP-Ox (E)**, and **1j (F)** in JNK3 (PDB code 1PMV). H-bonds are shown in blue dashed lines. Residues within 3 Å of each pose are visible.

In order to gain insight into interactions of the investigated compounds and explain some observations made in structure-activity relationship analysis, we performed molecular docking of **TRYP-Ox** (Schepetkin et al., 2019), **1c**, **1f**, **1i**, **1j**, and **1o** into the JNK3 binding site (PDB: 1PMV) using Molegro Virtual Docker (MVD) software. Note that these compounds exist as mixtures of *Z* and *E* isomers with respect to the exocyclic C=N bond. These geometric isomers are prone to interconversion (Dugave and Demange, 2003; Blanco et al., 2009). Therefore, we obtained docking poses for both *Z* and *E* isomers. The best docking poses are shown in Figure 4, and key interactions of the compounds with the kinase residues are presented in Table 2.

**TABLE 2:** MVD docking scores (DS) for the best docking poses, absolute differences ΔDS between *Z* and *E* isomers, and key interactions with the binding site of JNK3 (PDB: 1PMV) for compounds **1c**, **1f**, **1i**, **1o**, **1j**, and **TRYP-Ox**.

Compd.	JNK3 K_d_ (µM)^a^	DS (units)	ΔDS (units)	Characteristics of binding to JNK3
*Z* 1c	0.85 ± 0.01	−50.03	4.39	HB: Asn152 (amide oxygen)
				VdW: Ile70 (pDS = −14.56)
*E* 1f	N.B.	−12.85	44.99	HB: Lys93 (oxime oxygen and nitrogen atoms)
				VdW: Leu206 (pDS = −13.66)
*Z* 1i	1.5 ± 0.1	−37.26	31.63	HB: Lys93 (oxime oxygen, weak; nitrogen atoms in pyridine and tryptanthrin moieties)
				VdW: Val78 (pDS = −14.54)
*E 1j*	0.18 ± 0.04	−97.78	24.33	HB: Lys93 (heterocycle nitrogen, oxime nitrogen and oxygen); Gly76 (acetyl oxygen); Gln75 (acetyl oxygen, weak)
				VdW: Val78 (pDS = −17.36)
*E*-1o	0.17 ± 0.03	−60.71	9.01	HB: Lys93 (semicarbazone oxygen); Ser72 (semicarbazone NH_2_, weak); Asn152 (heterocycle nitrogen, weak)
				VdW: Val78 (pDS = −12.46)
*Z* TRYP-Ox	0.275 ± 0.21	−62.22	27.90	HB: Asp207 (OH group)
				VdW: Leu206 (pDS = −15.51)

a Binding affinity measured on a mixture of isomers. N.B., no JNK binding at concentrations ≤ 30 μM. HB, hydrogen bond; VdW, strong van der Waals interaction; pDS, partial docking score for van der Waals interaction.

**FIGURE 5:**
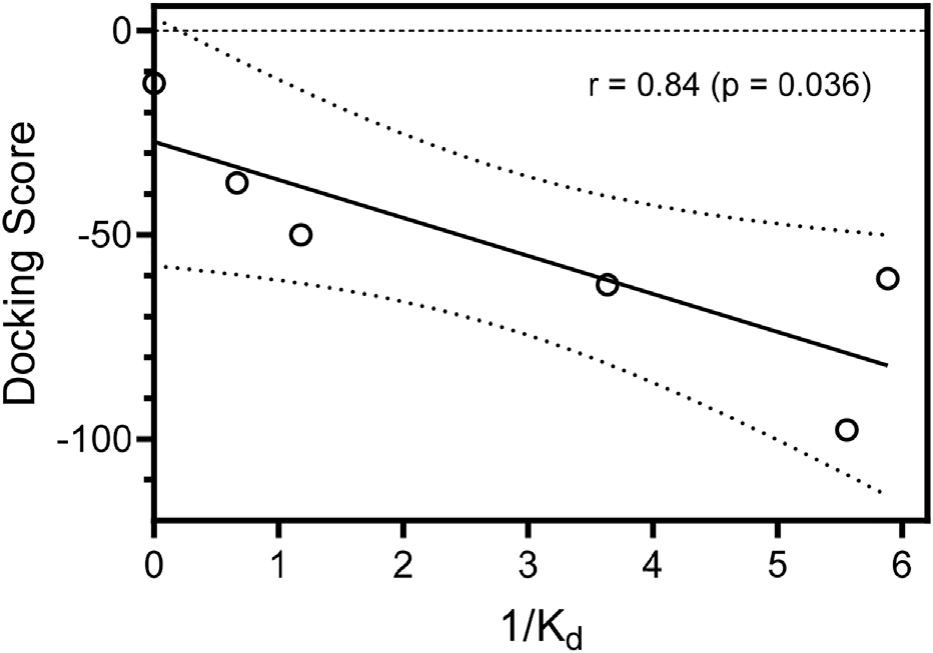
Correlation of docking scores for the best docking poses of the compounds in JNK3 and binding affinities (K_d_) with JNK3. Binding affinities are represented as inverse (1/K_d_) values. Dashed lines indicate area of the 95% confidence band.

The MolDock docking scores (DS) differ by 4.4−45 units between the isomeric oxime structures. For compounds **1f**, **1j**, and **1o** bound to JNK3, the lower (more negative) DS corresponds to the *E* isomer, while in the other cases, compounds bound to the kinase binding site preferably in the *Z* isomer form. We benchmarked our docking scores against measured binding affinities with JNK3 and found a significant correlation (r = 0.84; *p* = 0.036) between experimentally and computationally derived data (Figure 5).

*O*-Substitution of **TRYP-Ox** with an allyl group (**1c**) led to a docking pose with a H-bonding interaction of the ligand with JNK3 via the amide carbonyl group of the tryptanthrin fragment with the allyl moiety located in a hydrophobic region (Figure 4A). This hydrophobic pocket can accommodate small linear alkyl groups (**1a**-**c**) with similar efficiency; however, benzyl groups (**1f**) were too large, leading to a change in binding pose and loss of binding affinity. Interestingly, the isosteric (2-pyridyl)methyl group (**1i**) exhibited modest binding to JNK3, and the docking results indicated H-bonding between Lys93 and the pyridyl nitrogen (Figure 4C). Introducing a semicarbazone fragment to the oxime group of **TRYP-Ox** brings additional H-bond donors and acceptors into molecule **1o**. On the interaction with JNK3, this fragment was H-bonded to Lys93 via the semicarbazone oxygen atom. Additionally, weak H-bonds to Ser72 and Asn152 were formed, with participation of the NH_2_ group and heterocyclic nitrogen atom (Figure 4D). These interactions, along with a noticeable van der Waals attraction between the ligand and Val78 (Table 2), led to an increased affinity with JNK3 (Table 1). Of note, these additional polar interactions, which led to the increased binding of compound **1o**, resulted in a different pose to that of the unsubstituted oxime **TRYP-Ox** (Figure 4E). The most negative DS value corresponded to compound **1j**, which is anchored within the binding site by several H-bonds (Figure 4F).

JNKs are directly involved in controlling regulation of NF-κB/AP-1 transcriptional activity; therefore, drug discovery efforts have focused on the development of JNK inhibitors for treatment of inflammatory diseases (Bennett et al., 2003; Wagner and Laufer, 2006; Jung et al., 2010). One of the targets of activated JNKs is c-Jun, which is specifically phosphorylated on Ser63 and/or Ser73, making this protein capable of binding AP-1 sites in the nucleus (Hibi et al., 1993). Although AP-1 and NF-κB are regulated by different signaling pathways, cross-talk between these pathways occurs, mediated in part by the ability of certain Jun and Fos family proteins to interact with the p65 subunit of NF-κB (Fujioka et al., 2004). Here, we report novel and potent JNK inhibitors with a trypthantrin scaffold that had high JNK1-3 binding affinity and inhibited LPS-induced NF-κB/AP-1 transcription activity in THP-1Blue cells and IL-6 production in MonoMac-6 cells. The present work supports our previous studies suggesting that trypthantrin is a good scaffold for the development JNK inhibitors (Schepetkin et al., 2012; Schepetkin et al., 2015). The trypthantrin molecule has a rigid flat aromatic ring structure. Other tri- and tetracyclic planar fragments have also been reported as kinase inhibitor scaffolds for Aurora A kinase (Warner et al., 2006) and JNK (Bennett et al., 2001). In general, flat ring structures have been identified as kinase-specific privileged structures; i.e., compounds containing these fragments are enriched for kinase targets, compared with other target classes (Posy et al., 2011). Thus, although oxime side groups may contribute important interactions in the JNK binding site (Schepetkin et al., 2012; Schepetkin et al., 2015; Schepetkin et al., 2019; Liakhov et al., 2021; Schepetkin et al., 2021), the tetracyclic nucleus seems to be responsible for proper ligand positioning. Docking experiments performed in the present study and in our previous work (Schepetkin et al., 2019) show that the tryptanthrin nucleus stipulates good complementarity of the ligands to the JNK1-3 binding sites with similar orientations of the tetracyclic moiety. Comparison of docking poses of the most potent JNK inhibitors (**TRYP-Ox**, **1j**, **1o**) with the published coordinates of ATP (Xie et al., 1998) showed that the oxime moiety of the compounds is positioned in an orientation similar to that of the ATP purine base, which is anchored deep in the ATP-binding site among Gly71, Ser72, Gly73, Gln75, Val78, Lys93, Leu148, Met149, Asp150, Ala151, Asn152, Ser193, Asn194, Ile195, Val196, Leu206, and Asp207.

## 3 Conclusion

In summary, fifteen novel analogs of **TRYP-Ox** were synthesized and characterized. The reactions of ketone oximation and oxime alkylation were investigated based on this heterocyclic system. Compounds **1j**,**k**,**l** had high affinity for JNK1-3 (K_d_ < 1 μM) and also potently inhibited LPS-induced nuclear NF-κB/AP-1 activation and IL-6 production in human monocytic cells. Our molecular modeling showed that compounds **1c**, **1i**, and **TRYP-Ox** bound to JNK3, preferably as *Z* isomers, while for **1f**, **1j**, and **1o**, the docking poses of *E* isomers were characterized by more negative docking scores. Differences in the JNK3 binding affinities of **TRYP-Ox** and its derivatives can be well explained by H-bonding patterns of the ligands and magnitudes of their docking scores.

## 4 Experimental section

### 4.1 Chemistry

Tryptanthrin was purchased from Combi-Blocks (San Diego, CA, United States). All the other starting reagents were purchased from Sigma-Aldrich. The chemicals were of analytical grade and used without further purification. Reaction progress was monitored by thin-layer chromatography (TLC) with UV detection using pre-coated silica gel F254 (Merck). Melting points (m.p.) were determined using an electrothermal Mel-Temp capillary melting point apparatus. HPLC-MS analysis was performed on a Zorbax Eclipse Plus C18 2.1 mm × 50 mm 1.8 micron. Elemental analysis was performed with a Carlo Erba instrument. IR spectra were recorded on a FT-IR spectrometer Nicolet 5700 with KBr pellets. NMR spectra were recorded on a Bruker Avance III HD instrument (operating frequency ^1^H–400 MHz; ^13^C–100 MHz). Purity of the compounds, according to the NMR data, were at least 98%.

#### 4.1.1 General procedure of the tryptanthrin oximation by O-alkyl-hydroxylamine hydrochlorides and hydrazines

A mixture of tryptanthrin (**TRYP**) (0.5 mmol) and substituted hydroxylamine or hydrazine (0.7 mmol) in MeOH (10 ml) were refluxed for 3–8 h and monitored by TLC. The mixture was then cooled and poured into water (100 ml). The resulting precipitate was filtered, washed with water, and recrystallized from EtOH to give **1a-d**, **f-h**, **m-o** as colorless or flaxen solids.

#### 4.1.2 General procedure of alkylation of tryptanthrin oximes

Compounds **1e** and **1i** were synthesized as described previously (Schepetkin et al., 2019). To a suspension of **TRYP-Ox** (1.0 mmol) and Na_2_CO_3_ (0.127 g, 1.2 mmol) in DMSO (5 ml), was added dropwise a solution of alkyl bromide (1.5 mmol) in DMSO (5 ml). The mixture was stirred overnight at room temperature and poured into 200 ml of water. The resulting precipitate was filtered and recrystallized from EtOH.

#### 4.1.3 General procedure for the synthesis of TRYP-Ox salts (1k and 1l)

Compounds **1k** and **1l** were prepared from **TRYP-Ox** (0.263 g, 1.0 mmol) by treatment with an excess of corresponding LiOH (36 mg, 1.5 mmol) or NaOH (60 mg, 1.5 mmol) in refluxing MeOH (10 ml) for 3 h (TLC monitoring). After cooling the precipitate was filtered and recrystallized from EtOH.

##### 4.1.3.1 6-(methoxyimino)indolo[2,1-b]quinazolin-12(6H)-one (1a)

Yield 73%, a flaxen solid. M.p. 218°C. ^1^H NMR (CDCl_3_), δ, ppm: 8.64 (1H, d, *J* = 8.1 Hz), 8.41 (1H, dd, *J* = 8.0, 1.6 Hz), 8.28 (1H, d, *J* = 7.6 Hz), 7.96 (1H, dd, *J* = 8.1, 1.2 Hz), 7.79 (1H, ddd, *J* = 8.3, 7.2, 1.6 Hz), 7.59 (1H, td, *J* = 8.0, 1.2 Hz), 7.55 (1H, ddd, *J* = 8.0, 7.5, 1.1 Hz), 7.37 (1H, td, *J* = 7.7, 1.1 Hz), 4.41 (3H, s). ^13^C NMR (CDCl_3_), δ, ppm: 159.2, 148.1, 147.2, 144.7, 140.2, 134.8, 133.1, 129.0, 128.3, 128.1, 127.2, 126.9, 122.2, 119.0, 117.3, 65.2. Found, %: C 69.60, H 3.97, N 15.30. C_16_H_11_N_3_O_2_. Calculated, %: C 69.31, H 4.00, N 15.15. HRMS (ESI-TOF) *m/z*: [M + H]^+^ Calcd. for C_16_H_12_N_3_O_2_ 278.0930, found 278.0919.

##### 4.1.3.2 6-(ethoxyimino)indolo[2,1-b]quinazolin-12(6H)-one (1b)

Yield 89%, a flaxen solid. M.p. 117°C. ^1^H NMR (CDCl_3_), δ, ppm: 8.65 (1H, d, *J* = 8.1 Hz), 8.41 (1H, dd, *J* = 8.0, 1.6 Hz), 8.30 (1H, d, *J* = 7.7 Hz), 7.96 (1H, dd, *J* = 8.2, 1.2 Hz), 7.79 (1H, ddd, *J* = 8.3, 7.1, 1.6 Hz), 7.58 (1H, td, *J* = 8.0, 1.2 Hz), 7.55 (1H, ddd, *J* = 8.1, 7.0, 1.3 Hz), 7.37 (1H, td, *J* = 7.7, 1.0 Hz), 4.68 (2H, q, *J* = 7.1 Hz), 1.52 (3H, t, *J* = 7.1 Hz). ^13^C NMR (CDCl_3_), δ, ppm: 159.3, 148.2, 147.3, 144.5, 140.1, 134.7, 132.9, 129.0, 128.2, 128.0, 127.2, 126.9, 122.1, 119.1, 117.2, 73.5, 14.9. Found, %: C 70.33, H 4.31, N 14.62. C_17_H_13_N_3_O_2_. Calculated, %: C 70.09, H 4.50, N 14.42. HRMS (ESI-TOF) *m/z*: [M + H]^+^ Calcd. for C_17_H_14_N_3_O_2_ 292.1086, found 292.1079.

##### 4.1.3.3 6-((allyloxy)imino)indolo[2,1-b]quinazolin-12(6H)-one (1c)

Yield 86%, a flaxen solid. M.p. 163°C. ^1^H NMR (CDCl_3_), δ, ppm: 8.65 (1H, d, *J* = 8.1 Hz), 8.42 (1H, dd, *J* = 7.9, 1.6 Hz), 8.31 (1H, d, *J* = 7.7 Hz), 7.96 (1H, d, *J* = 7.8 Hz), 7.80 (1H, ddd, *J* = 8.4, 7.2, 1.6 Hz), 7.60 (1H, td, *J* = 7.9, 1.3 Hz), 7.56 (1H, ddd, *J* = 8.1, 7.0, 1.0 Hz), 7.38 (1H, td, *J* = 7.7, 1.1 Hz), 6.18 (1H, ddt, *J* = 17.2, 10.4, 5.9 Hz), 5.48 (1H, dd, *J* = 17.2, 1.5 Hz), 5.38 (1H, dd, *J* = 10.5, 1.3 Hz), 5.12 (2H, d, *J* = 5.9 Hz). ^13^C NMR (CDCl_3_), δ, ppm: 159.2, 148.1, 147.3, 145.0, 140.2, 134.8, 133.1, 132.8, 129.0, 128.4, 128.1, 127.2, 127.0, 122.2, 119.5, 119.1, 117.3, 76.8. Found, %: C 71.56, H 4.19, N 14.02. C_18_H_13_N_3_O_2_. Calculated, %: C 71.28, H 4.32, N 13.85. HRMS (ESI-TOF) *m/z*: [M + H]^+^ Calcd. for C_18_H_14_N_3_O_2_ 304.1086, found 304.1086.

##### 4.1.3.4 6-(tert-butoxyimino)indolo[2,1-b]quinazolin-12(6H)-one (1d)

Yield 50%, a flaxen solid. M.p. 229°C. *E*-isomer: ^1^H NMR (CDCl_3_), δ, ppm: 8.67 (1H, d, *J* = 8.1 Hz), 8.42 (1H, dd, *J* = 8.0, 1.6 Hz), 8.33 (1H, d, *J* = 7.6 Hz), 7.95 (1H, d, *J* = 8.1 Hz), 7.79 (1H, ddd, *J* = 8.3, 7.2, 1.6 Hz), 7.58 (1H, td, *J* = 8.0, 1.1 Hz), 7.57–7.52 (1H, m), 7.38 (1H, t, *J* = 7.9 Hz), 1.59 (s, 9H). ^13^C NMR (CDCl_3_), δ, ppm: 159.4, 148.4, 147.5, 144.0, 139.9, 134.6, 132.5, 128.9, 128.1, 127.8, 127.2, 126.8, 122.0, 119.5, 117.2, 83.8, 27.9. *Z*-isomer: ^1^H NMR (CDCl_3_), δ, ppm: 8.67 (1H, d, *J* = 8.1 Hz), 8.42 (1H, dd, *J* = 8.0, 1.6 Hz), 8.33 (1H, d, *J* = 7.6 Hz), 7.95 (1H, d, *J* = 8.1 Hz), 7.79 (1H, ddd, *J* = 8.3, 7.2, 1.6 Hz), 7.58 (1H, td, *J* = 8.0, 1.1 Hz), 7.57–7.52 (1H, m), 7.38 (1H, t, *J* = 7.9 Hz), 1.70 (s, 9H). ^13^C NMR (CDCl_3_), δ, ppm: 159.4, 148.4, 147.5, 144.0, 139.9, 134.4, 131.1, 128.5, 128.1, 127.7, 127.0, 126.8, 122.0, 119.5, 117.3, 83.8, 27.64. Found, %: C 71.68, H 5.26, N 13.28. C_19_H_17_N_3_O_2_. Calculated, %: C 71.46, H 5.37, N 13.16. HRMS (ESI-TOF) *m/z*: [M + H]^+^ Calcd. for C_19_H_18_N_3_O_2_ 320.1399, found 320.1404.

##### 4.1.3.5 2-(((12-oxoindolo[2,1-b]quinazolin-6(12H)-ylidene)amino)oxy)acetonitrile (1e)

Compound **2e** was synthesized according to the general procedure of alkylation from **TRYP-Ox** (0.263 g, 1.0 mmol) and 2-bromoacetonitrile (0.104 ml, 1.5 mmol). Yield 3% (10 mg), dark brown crystals. M.p. 224°C. ^1^H NMR (CDCl_3_), δ, ppm: 8.67 (1H, d, *J* = 8.1 Hz), 8.44 (1H, dd, *J* = 7.9, 1.5 Hz), 8.26 (1H, d, *J* = 7.7 Hz), 7.97 (1H, d, *J* = 7.8 Hz), 7.83 (1H, ddd, *J* = 8.3, 7.2, 1.5 Hz), 7.67 (1H, td, *J* = 7.8, 1.3 Hz), 7.61 (1H, ddd, *J* = 8.2, 7.2, 1.2 Hz), 7.41 (1H, ddd, *J* = 7.7, 7.7, 1.0 Hz), 5.24 (2H, s). ^13^C NMR (CDCl_3_), δ, ppm: 158.9, 147.6, 146.8, 145.7, 140.9, 135.1, 134.4, 133.1, 129.7, 129.2, 128.8, 127.3, 127.1, 122.4, 118.3, 117.5, 61.4. HRMS (ESI-TOF) *m/z*: [M + H]^+^ Calcd. for C_17_H_11_N_4_O_2_ 303.0882, found 303.0885.

##### 4.1.3.6 6-((benzyloxy)imino)indolo[2,1-b]quinazolin-12(6H)-one (1f)

Yield 96%, a flaxen solid. M.p. 206°C. *E*-isomer: ^1^H NMR (CDCl_3_), δ, ppm: 8.65 (1H, d, *J* = 8.1 Hz), 8.43 (1H, dd, *J* = 8.0, 1.6 Hz), 8.25 (1H, d, *J* = 8.1 Hz), 7.97 (1H, d, *J* = 7.8 Hz), 7.81 (1H, ddd, *J* = 8.5, 7.2, 1.5 Hz), 7.62–7.54 (2H, m), 7.51 (2H, d, *J* = 6.6 Hz), 7.46–7.38 (3H, m), 7.33 (1H, t, *J* = 7.7 Hz), 5.66 (2H, s). ^13^C NMR (CDCl_3_), δ, ppm: 159.3, 148.2, 147.3, 145.1, 140.2, 136.1, 134.8, 133.2, 129.0, 128.9, 128.8, 128.7, 128.5, 128.1, 127.3, 127.0, 122.2, 119.1, 117.2, 76.8. *Z*-isomer: ^1^H NMR (CDCl_3_), δ, ppm: 8.58 (1H, d, *J* = 8.1 Hz), 8.43 (1H, dd, *J* = 8.0, 1.6 Hz), 8.25 (1H, d, *J* = 8.1 Hz), 7.94 (1H, d, *J* = 7.8 Hz), 7.81 (1H, ddd, *J* = 8.5, 7.2, 1.5 Hz), 7.62–7.54 (2H, m), 7.51 (2H, d, *J* = 6.6 Hz), 7.46–7.38 (3H, m), 7.33 (1H, t, *J* = 7.7 Hz), 5.62 (2H, s). Found, %: C 75.02, H 4.02, N 12.01. C_22_H_15_N_3_O_2_. Calculated, %: C 74.78, H 4.28, N 11.89. HRMS (ESI-TOF) *m/z*: [M + H]^+^ Calcd. for C_22_H_16_N_3_O_2_ 354.123, found 354.1257.

##### 4.1.3.7 6-(((pentafluorophenyl)methoxy)imino)indolo[2,1-b]quinazolin-12(6H)-one (1g)

Yield 90%, a flaxen solid. M.p. 183°C. *E*-isomer: ^1^H NMR (CDCl_3_), δ, ppm: 8.63 (1H, d, *J* = 8.1 Hz), 8.41 (1H, dd, *J* = 8.0, 1.6 Hz), 8.14 (1H, d, *J* = 7.3 Hz), 7.96 (1H, d, *J* = 7.9 Hz), 7.81 (1H, t, *J* = 7.7 Hz), 7.59 (2H, t, *J* = 8.0 Hz), 7.35 (1H, t, *J* = 7.7 Hz), 5.73 (s, 2H). ^13^C NMR (CDCl_3_), δ, ppm: 159.1, 147.7, 147.5 – 147.1 and 143.7–143.3 (2 × m), 147.1, 146.3–145.9 and 143.7–143.2 (2 × m), 146.0, 140.5, 134.9, 139.2–138.8 and 136.7–136.3 (2 × m), 133.7, 129.1, 128.6, 128.4, 127.3, 127.0, 122.3, 118.8, 117.3, 66.1. *Z*-isomer: ^1^H NMR (CDCl_3_), δ, ppm: 8.55 (1H, d, *J* = 8.1 Hz), 8.41 (1H, dd, *J* = 8.0, 1.6 Hz), 8.14 (1H, d, *J* = 7.3 Hz), 7.92 (1H, d, *J* = 7.9 Hz), 7.77 (1H, d, *J* = 7.7 Hz), 7.55 (2H, t, *J* = 8.0 Hz), 7.35 (1H, t, *J* = 7.7 Hz), 5.64 (s, 2H). ^13^C NMR (CDCl_3_), δ, ppm: 159.0, 147.4, 147.5–147.1 and 143.7–143.3 (2 × m), 146.3–145.9 and 143.7–143.2 (2 × m), 144.0, 142.7, 139.8, 134.6, 139.2–138.8 and 136.7–136.3 (2 × m), 132.2, 128.1, 128.6, 128.4, 127.1, 126.7, 121.4, 118.8, 117.4, 65.7. HRMS (ESI-TOF) *m/z*: [M + H]^+^ Calcd. for C_22_H_11_F_5_N_3_O_2_ 444.0771, found 444.0766.

##### 4.1.3.8 6-(((4-nitrobenzyl)oxy)imino)indolo[2,1-b]quinazolin-12(6H)-one (1h)

Yield 95%, a flaxen solid. M.p. 219°C. *E*-isomer: ^1^H NMR (CDCl_3_), δ, ppm: 8.68 (1H, d, *J* = 8.1 Hz), 8.43 (1H, dd, *J* = 7.9, 1.6 Hz), 8.31–8.25 (3H, m), 7.95 (1H, d, *J* = 7.9 Hz), 7.81 (1H, ddd, *J* = 8.3, 7.2, 1.6 Hz), 7.68–7.56 (4H, m), 7.38 (1H, t, *J* = 7.6 Hz), 5.75 (2H, s). ^13^C NMR (CDCl_3_), δ, ppm: 159.1, 148.1, 147.8, 147.1, 146.0, 143.7, 140.5, 134.9, 133.7, 129.1, 128.7, 128.5, 128.4, 127.3, 127.1, 124.1, 122.3, 118.9, 117.5, 78.0. *Z*-isomer: ^1^H NMR (CDCl_3_), δ, ppm: 8.59 (1H, d, *J* = 8.1 Hz), 8.45 (1H, dd, *J* = 7.9, 1.6 Hz), 8.31–8.25 (3H, m), 7.95 (1H, d, *J* = 7.9 Hz), 7.81 (1H, ddd, *J* = 8.3, 7.2, 1.6 Hz), 7.68–7.56 (4H, m), 7.34 (1H, t, *J* = 7.6 Hz), 5.75 (2H, s). ^13^C NMR (CDCl_3_), δ, ppm: 159.0, 148.1, 147.8, 147.4, 147.0, 144.6, 139.8, 134.7, 132.3, 129.7, 129.2, 128.7, 128.3, 127.2, 126.7, 123.9, 121.4, 119.1, 117.5, 77.6. Found, %: C 66.59, H 3.42, N 14.24. C_22_H_14_N_4_O_4_. Calculated, %: C 66.33, H 3.54, N 14.06. HRMS (ESI-TOF) *m/z*: [M + H]^+^ Calcd. for C_22_H_15_N_4_O_4_ 399.1093, found 399.1116.

##### 4.1.3.9 6-((pyridin-2-ylmethoxy)imino)indolo[2,1-b]quinazolin-12(6H)-one (1i)

Compound **2i** was synthesized according to the general procedure of alkylation from **TRYP-Ox** (0.263 g, 1.0 mmol), 2-(bromomethyl)pyridine hydrobromide (0.3794 g, 1.5 mmol) and Na_2_CO_3_ (0.2861 g, 2.7 mmol). Yield 18% (64 mg), light green crystals. M.p. 199–200°C. ^1^H NMR (CDCl_3_), δ, ppm: 8.72–8.62 (2H, m), 8.42 (1H, d, *J* = 7.9 Hz), 8.36 (1H, d, *J* = 7.7 Hz), 7.95 (1H, d, *J* = 8.2 Hz), 7.85–7.74 (2H, m), 7.67–7.51 (3H, m), 7.37 (1H, dd, *J* = 7.7, 7.7 Hz), 7.32 (1H, dd, *J* = 7.6, 5.0 Hz), 5.82 (2H, s). ^13^C NMR (CDCl_3_), δ, ppm 159.2, 156.2, 148.8, 148.0, 147.2, 145.9, 140.4, 138.0, 134.9, 133.5, 129.0, 128.7, 128.3, 127.3, 127.1, 123.5, 122.6, 122.3, 119.0, 117.3, 79.4. HRMS (ESI-TOF) *m/z*: [M + H]^+^ Calcd. for C_21_H_15_N_4_O_2_ 355.1195, found 355.1215.

##### 4.1.3.10 The synthesis of 6-(acetoxyimino)indolo[2,1-b]quinazolin-12(6H)-one (1j)

Prepared from **TRYP-Ox** (80.0 mg, 0.3 mmol) and acetic anhydride (0.468 ml, 4.5 mmol) in pyridine (5 ml) at 0°C. Then the mixture was poured into the water (150 ml), filtered and recrystallized from EtOH. Yield 78%, a colorless solid. M.p. 262°C. *E*-isomer: ^1^H NMR (CDCl_3_), δ, ppm: 8.67 (1H, d, *J* = 8.1 Hz), 8.42 (1H, dd, *J* = 7.8, 1.5 Hz), 8.34 (1H, d, *J* = 7.4 Hz), 7.97 (1H, dd, *J* = 8.2, 1.2 Hz), 7.83 (1H, ddd, *J* = 8.2, 7.2, 1.6 Hz), 7.69 (1H, ddd, *J* = 7.9, 7.9, 1.3 Hz), 7.61 (1H, ddd, *J* = 8.1, 7.2, 1.2 Hz), 7.43 (1H, ddd, *J* = 7.7, 7.7, 1.0 Hz), 2.50 (3H, s). ^13^C NMR (CDCl_3_), δ, ppm 167.7, 158.9, 149.9, 147.2, 146.9, 141.6, 135.1, 135.1, 129.6, 129.5, 129.0, 127.4, 127.2, 122.6, 118.2, 117.7, 19.6. *Z*-isomer: ^1^H NMR (CDCl_3_), δ, ppm: 8.58 (1H, d, *J* = 8.1 Hz), 8.42 (1H, dd, *J* = 7.8, 1.5 Hz), 8.34 (1H, d, *J* = 7.4 Hz), 8.04 (1H, d, *J* = 8.2 Hz), 7.83 (1H, ddd, *J* = 8.2, 7.2, 1.6 Hz), 7.69 (1H, ddd, *J* = 7.9, 7.9, 1.3 Hz), 7.61 (1H, ddd, *J* = 8.1, 7.2, 1.2 Hz), 7.39 (1H, dd, *J* = 7.7, 7.7 Hz), 2.47 (3H, s). ^13^C NMR (CDCl_3_), δ, ppm 168.2, 158.7, 149.9, 147.2, 146.9, 141.9, 134.9, 134.1, 130.0, 129.8, 129.0, 127.4, 127.0, 123.2, 118.2, 117.5, 19.6. Found, %: C 69.62, H 3.77, N 14.83. C_17_H_11_N_3_O_3_. Calculated, %: C 69.31, H 4.00, N 15.15. HRMS (ESI-TOF) *m/z*: [M + H]^+^ Calcd. for C_17_H_12_N_3_O_3_ 306.0879, found 306.0898.

##### 4.1.3.11 6-(hydroximino)indolo[2,1-b]quinazolin-12(6H)-one lithium salt (1k)

Yield 34%, colorless crystals. M.p. 399°C. ^1^H NMR (DMSO-d_6_), δ, ppm: 8.64–8.58 (2H, m), 8.25 (1H, dd, *J* = 7.9, 1.6 Hz), 7.74 (1H, ddd, *J* = 8.4, 6.9, 1.6 Hz), 7.67 (1H, dd, *J* = 8.3, 1.2 Hz), 7.35 (1H, ddd, *J* = 8.0, 6.8, 1.3 Hz), 7.31–7.24 (2H, m). ^13^C NMR (DMSO-d_6_), δ, ppm: 159.7, 152.3, 149.5, 145.6, 133.7, 132.3, 126.5, 126.3, 126.2, 124.5, 122.9, 119.5, 119.5, 118.5, 115.4. Found, %: C 66.67, H 2.98, N 15.28. C_15_H_8_LiN_3_O_2_. Calculated, %: C 66.93, H 3.00, N 15.61.

##### 4.1.3.12 6-(hydroximino)indolo[2,1-b]quinazolin-12(6H)-one sodium salt (1l)

Yield 88%, colorless crystals. M.p. >400°C, decomp. ^1^H NMR (DMSO-d_6_), δ, ppm: 8.59 (1H, d, *J* = 8 Hz), 8.57 (1H, dd, *J* = 7.9, 1.4 Hz), 8.26 (1H, dd, *J* = 8.0, 1.5 Hz), 7.79 (1H, ddd, *J* = 8.3, 6.8, 1.6 Hz), 7.74 (1H, d, *J* = 7.4 Hz), 7.45–7.35 (2H, m), 7.32 (1H, ddd, *J* = 7.5, 7.5, 1.3 Hz). ^13^C NMR (DMSO-d_6_), δ, ppm: 159.4, 151.3, 148.8, 145.0, 134.1, 134.0, 126.9, 126.4, 126.4, 124.1, 121.6, 121.6, 119.5, 119.5, 115.6. Found, %: C 63.42, H 2.68, N 14.70. C_15_H_8_NaN_3_O_2_. Calculated, %: C 63.16, H 2.83, N 14.73. HRMS (ESI-TOF) *m/z*: [M + H]^+^ Calcd. for C_15_H_9_N_3_NaO_2_ 286.05925, found 286.0592.

##### 4.1.3.13 6-hydrazonoindolo[2,1-b]quinazolin-12(6H)-one (1m)

Yield 93%, an orange solid. M.p. >400°C with decomposition. ^1^H NMR (CDCl_3_), δ, ppm: 11.21 (2H, s), 8.63 (1H, d, *J* = 8.1 Hz), 8.46 (1H, d, *J* = 7.9 Hz), 8.04 (1H, d, *J* = 8.0 Hz), 7.92 (1H, d, *J* = 7.7 Hz), 7.85 (1H, t, *J* = 7.0 Hz), 7.68 (1H, t, *J* = 8.1 Hz), 7.44 (1H, t, *J* = 7.5 Hz), 7.37 (1H, td, *J* = 7.9, 0.9 Hz). ^13^C NMR (CDCl_3_), δ, ppm: 159.1, 147.3, 146.1, 138.5, 135.3, 134.5, 130.9, 128.5, 127.7, 127.5, 126.5, 125.6, 119.0, 118.1, 117.1. Found, %: C 68.83, H 3.66, N 21.62. C_15_H_10_N_4_O. Calculated, %: C 68.69, H 3.84, N 21.36. HRMS (ESI-TOF) *m/z*: [M + H]^+^ Calcd. for C_15_H_11_N_4_O 263.0933, found 263.0934.

##### 4.1.3.14 6-(2-phenylhydrazono)indolo[2,1-b]quinazolin-12(6H)-one (1n)

Yield 90%, an orange solid. M.p. 223°C. ^1^H NMR (CDCl_3_), δ, ppm: 13.48 (1H, s), 8.58 (1H, d, *J* = 7.7 Hz), 8.46 (1H, d, *J* = 7.9 Hz), 7.89 (1H, d, *J* = 7.5 Hz), 7.83–7.80 (2H, m), 7.55 (1H, dt, *J* = 8.2, 4.2 Hz), 7.50–7.36 (6H, m), 7.11 (1H, t, *J* = 7.2 Hz). ^13^C NMR (CDCl_3_), δ, ppm: 159.0, 147.0, 146.2, 142.8, 138.4, 137.2, 135.3, 134.5, 129.7, 128.5, 127.5, 127.5, 127.5, 126.5, 123.7, 121.5, 119.2, 117.1, 114.7. Found, %: C 74.79, H 3.98, N 16.42. C_21_H_14_N_4_O. Calculated, %: C 74.54, H 4.17, N 16.56. HRMS (ESI-TOF) *m/z*: [M + H]^+^ Calcd. for C_21_H_15_N_4_O 339.1246, found 339.1263.

##### 4.1.3.15 2-(12-oxoindolo[2,1-b]quinazolin-6(12H)-ylidene)hydrazinecarboxamide (1o)

Yield 91%, a yellow solid. M.p. 266°C. ^1^H NMR (CDCl_3_), δ, ppm: 12.62 (1H, s), 8.68 (1H, d, *J* = 8.1 Hz), 8.49 (1H, dd, *J* = 8.1, 1.5 Hz), 8.10 (1H, d, *J* = 8.0 Hz), 7.90–7.83 (2H, m), 7.71–7.68 (1H, m), 7.55 (1H, td, *J* = 7.9, 1.3 Hz), 7.43 (1H, td, *J* = 7.5, 0.7 Hz). ^13^C NMR (CDCl_3_), δ, ppm: 159.0, 155.8, 147.4, 146.4, 141.9, 138.5, 135.1, 134.6, 131.3, 130.0, 128.5, 127.7, 126.8, 125.6, 120.5, 117.5. Found, %: C 63.22, H 3.51, N 22.62. C_16_H_11_N_5_O_2_. Calculated, %: C 62.95, H 3.63, N 22.94. HRMS (ESI-TOF) *m/z*: [M + H]^+^ Calcd. for C_16_H_12_N_5_O_2_ 306.0991, found 306.1006.

### 4.2 Methods for biological analysis

#### 4.2.1 Kinase K_d_ determination

Selected compounds were submitted for dissociation constant (K_d_) determination using the KINOMEscan platform (Eurofins Pharma Discovery, San Diego, CA, United States), as described previously (Karaman et al., 2008). In brief, kinases were produced and displayed on T7 phage or expressed in HEK-293 cells. Binding reactions were performed at room temperature for 1 h, and the fraction of kinase not bound to test compound was determined by capture with an immobilized affinity ligand and quantified by quantitative polymerase chain reaction. Primary screening at fixed concentrations of compounds was performed in duplicate. For dissociation constant K_d_ determination, a 12-point half-log dilution series (a maximum concentration of 33 μM) was used. Assays were performed in duplicate, and their average mean value is displayed.

#### 4.2.2 Cell culture

All cells were cultured at 37°C in a humidified atmosphere containing 5% CO_2_. THP-1Blue cells obtained from InvivoGen (San Diego, CA, United States) were cultured in RPMI 1640 medium (Mediatech Inc., Herndon, VA, United States) supplemented with 10% (v/v) fetal bovine serum (FBS), 4.5 g/L glucose, 100 μg/ml streptomycin, 100 U/mL penicillin, 100 μg/ml phleomycin (Zeocin), and 10 μg/ml blasticidin S. Human monocyte-macrophage MonoMac-6 cells (Deutsche Sammlung von Mikroorganismen und Zellkulturen GmbH, Braunschweig, Germany) were grown in RPMI 1640 medium supplemented with 10% (v/v) FBS, 10 μg/ml bovine insulin, 100 μg/ml streptomycin, and 100 U/mL penicillin.

#### 4.2.3 Analysis of AP-1/NF-κB activation

Activation of AP-1/NF-κB was measured using an alkaline phosphatase reporter gene assay in THP1-Blue cells. Human monocytic THP-1Blue cells are stably transfected with a secreted embryonic alkaline phosphatase gene that is under the control of a promoter inducible by AP-1/NF-κB. THP-1Blue cells (2 × 10^5^ cells/well) were pretreated with test compound or DMSO (1% final concentration) for 30 min, followed by addition of 250 ng/ml LPS (from *Escherichia coli* K-235; Sigma Chemical Co., St. Louis, MO, United States) for 24 h, and alkaline phosphatase activity was measured in cell supernatants using QUANTI-Blue mix (InvivoGen) with absorbance at 655 nm and compared with positive control samples (LPS). The concentrations of compound that caused 50% inhibition of the AP-1/NF-κB reporter activity (IC_50_) were calculated.

#### 4.2.4 Cytokine analysis

Human IL-6 ELISA kit (BD Biosciences, San Jose, CA, United States) was used to confirm the inhibitory effect of selected compounds on IL-6 production. MonoMac-6 cells were plated in 96-well plates at a density of 2 × 10^5^ cells/well in culture medium supplemented with 3% (v/v) endotoxin-free FBS. Cells were pretreated with test compound or DMSO (1% final concentration) for 30 min, followed by addition of 250 ng/ml LPS for 24 h. IC_50_ values for IL-6 production were calculated by plotting the percentage inhibition against the logarithm of inhibitor concentration (at least five points). Multiplex human cytokine ELISA kit from Anogen (Mississauga, ON, Canada) was used to evaluate various cytokines (IL-1α, IL-1β, IL-6, GM-CSF, MCP-1, IFNγ, and TNF) in the supernatants of MonoMac-6 cells.

#### 4.2.5 Cytotoxicity assay

Cytotoxicity was analyzed with a CellTiter-Glo Luminescent Cell Viability Assay Kit from Promega (Madison, WI, United States), according to the manufacturer’s protocol. Cells were treated with the compound under investigation and cultivated for 24 h. After treatment, the cells were allowed to equilibrate to room temperature for 30 min, substrate was added, and the luminescence measured using a Fluoroscan Ascent FL (Thermo Fisher Scientific, Waltham, MA, United States). The cell IC_50_ values were calculated by plotting the percentage inhibition against the logarithm of inhibitor concentration (at least five points).

#### 4.2.6 Western blotting

MonoMac-6 monocytic cells (10^7^ cells) were incubated with different concentrations of compound **1j** (final DMSO concentration of 0.5%) for 30 min at 37°C and then treated with LPS (250 ng/ml) or buffer for another 30 min at 37°C. Cells were washed twice with ice-cold phosphate buffer solution (pH 7.4), and cell lysates were prepared using lysis buffer (Cell Signaling Technology, Danvers, MA, United States). Cell lysates were separated on ExpressPlus 10% PAGE Gels (GenScript, Piscataway, NJ, United States) using TRIS-MOPS running buffer and transferred to nitrocellulose membranes. The blots were blocked overnight at 4°C in TRIS buffer (pH 7.4) + 0.1% Tween-20 (TBST) + 2.5% bovine serum albumin and probed with antibodies against phospho-c-Jun (Ser63) (Cell Signaling Technology), followed by horseradish peroxidase-conjugated secondary antibody (Cell Signaling Technology), and the blots were developed using Super-Signal West Femto chemiluminescent substrate (Thermo Fisher Scientific) and visualized with a FluorChem FC2 imaging system (Alpha Innotech Corporation, San Leandro, CA, United States). For measurement of total c-Jun signal, we stripped and reprobed the same Western blots that were used for phospho-c-Jun blots. Briefly, the membranes were washed 4 times for 5 min with TBST, incubated for 30 min at 50°C in TRIS buffer (pH 6.3) + 2% sodium dodecyl sulfate + 0.63% β-mercaptoethanol and then washed 6 times for 5 min each wash in TBST. The membranes were blocked again and probed for total c-Jun, followed by horseradish peroxidase-conjugated secondary antibody (both reagents from Cell Signaling Technology), developed, and visualized as described above. Quantitation of the luminescent signals were performed using AlphaView software.

### 4.3 Molecular docking

Geometries of JNK3 protein was obtained by downloading crystal structures from the Protein Data Bank (PDB entry code 1PMV) into Molegro software (Molegro ApS, Aarhus, Denmark). All solvent molecules were removed. A search space was chosen for each of the receptors as a sphere centered on co-crystallized ligand present in the corresponding PDB structure. Radius of the sphere was equal to 10 Å. The sphere completely encompassed the co-crystallized ligand and the binding site. Side chains of all amino acid residues of a receptor within the corresponding sphere were regarded as flexible during docking. The number of such residues was equal to 39. The flexible residues were treated with default settings of “Setup Sidechain Flexibility” tool in Molegro, and a softening parameter of 0.7 was applied during flexible docking, according to the standard protocol using the Molegro Virtual Docker 6.0 (MVD) program. Before docking, structures of compounds were pre-optimized using HyperChem software (HyperCube, Gainesville, FL, United States) with the MM+ force field and saved in Tripos MOL2 format (Tripos, St. Louis, MO, United States). The ligand structures were imported into MVD. The options “Create explicit hydrogens,” “Assign charges (calculated by MVD),” and “Detect flexible torsions in ligands” were enabled during importing. Appropriate protonation states of the ligands were also automatically generated at this step. Each ligand was subJected to 30 docking runs with respect to a given receptor structure using MVD software. The docking pose with the lowest MolDock docking score (Thomsen et al., 2006) was selected for each ligand and analyzed using the built-in tools of MVD.

## Data Availability

The raw data supporting the conclusion of this article will be made available by the authors, without undue reservation.
